# Complete genome sequence of *Fundidesulfovibrio* sp. strain YSD1, an electroactive bacterium isolated from estuarine sediment

**DOI:** 10.1128/mra.01193-25

**Published:** 2026-01-14

**Authors:** Keisuke Tomita, Mizuki Kato, Kazuya Watanabe

**Affiliations:** 1 School of Life Sciences, Tokyo University of Pharmacy and Life Sciences 13115, Hachioji, Tokyo, Japan; California State University San Marcos, San Marcos, California, USA

**Keywords:** electroactive bacteria, estuarine, Fundidesulfovibrio

## Abstract

*Fundidesulfovibrio* sp. strain YSD1 was isolated from the anode of a sediment microbial fuel cell constructed with estuarine mud. Here, we report a complete genome sequence of YSD1, which will expand our knowledge of the diversity and ecology of electroactive bacteria and highlight potential applications in microbial electrochemical technologies.

## ANNOUNCEMENT

Electroactive microorganisms (EAMs), which can exchange electrons with extracellular conductive materials, such as minerals or electrodes, play a crucial role in geochemical cycling of various elements by catalyzing redox reactions. Additionally, the electrical output from their metabolism can be utilized for various microbial electrochemical technologies (1). Many EAMs have been isolated to date (2, 3); however, the overall diversity remains poorly understood. To isolate novel EAMs, we operated a sediment microbial fuel cell (sMFC) using estuarine sediment from the Kyobashi River (34.37 N 132.46 E, Hiroshima, Japan). After the operation, the anode was removed from the sMFC and suspended in DSMZ826 medium (German Collection of Microorganisms and Cell Cultures) in an anaerobic chamber. Serial dilutions of the suspension were spread onto DSMZ826 agar plates and incubated for 5 days at 30°C. By repeating single colony isolation, strain YSD1 was isolated. This strain achieved current densities as high as 0.25 mA cm^−2^ per projected anode area similar to other high-current-producing EAMs, such as *Shewanella oneidensis* MR-1 (0.2 mA cm^−2^), in bioelectrochemical cells described elsewhere (4). This motivated us to determine the genome sequence of YSD1.

For DNA isolation, YSD1 was anaerobically cultured in DSMZ826 medium for 5 days at 37°C. Genomic DNA was extracted using the FavorPrep Tissue Genomic DNA Extraction Kit (FAVORGEN, Ping Tung, Taiwan) and further purified with 1.2× ProNex Beads (Promega, Madison, WI, USA). No shearing or size selection of DNA was performed. The sequencing library was constructed using a SMRTbell Prep Kit 3.0 (Pacific Biosciences, Menlo Park, CA, USA) and a SMRTbell gDNA Sample Amplification Kit (Pacific Biosciences). After primer annealing and polymerization using a Revio Polymerase kit, sequencing was performed using a Revio system (Pacific Biosciences). Overhang adapter sequences were trimmed from raw reads using SMRT Link (ver. 13.1.0.221970) (Pacific Biosciences) to generate subreads. Circular consensus sequences (CCS) were generated by aligning the subreads, and CCS with quality values below 20 were removed to generate HiFi reads. Ultra-low PCR adapters were removed from HiFi reads using lima (ver. 2.12.0) (https://github.com/pacificbiosciences/barcoding), and PCR duplicates were removed using pbmarkdup (ver. 1.0.3) (https://github.com/PacificBiosciences/pbmarkdup). Finally, HiFi reads less than 1,000 bp were removed using Filtlong (ver. 0.2.1) (https://github.com/rrwick/Filtlong). The remaining HiFi reads (33,761 reads) were *de novo* assembled using Flye (ver. 2.9.3-b1797) to generate circular contigs (5). The genome was rotated so that the *dnaA* gene appeared first. DFAST (ver. 1.3.6) was used for gene annotation and average nucleotide identity (ANI) calculation (6–8). Genome completeness and contamination were assessed by CheckM2 (ver. 1.0.1) (9). Default parameters were used for all software.

The assembly of HiFi reads by Flye yielded a single circular contig (Table 1). The 16S rRNA sequence of strain YSD1 was most closely related to that of *Fundidesulfovibrio terrae* SG127^T^ (GCA_022808915.1) (10), with 98.38% identity in the BLAST search (ver. 2.17.0) using Core nucleotide BLAST database (last updated in 2025/10/05) as a reference, and the ANI of these two strains was 83.57%. Although a formal description awaits further taxonomic characterization, YSD1 appears to represent a distinct lineage within the genus *Fundidesulfovibrio*.

**TABLE 1:** Sequencing statistics and genomic features of strain YSD1

	YSD1
Sequencing results	
Number of reads	40,015
Total read length (bp)	249,709,875
Read N_50_ (bp)	6,798
Assembly results	
Total length (bp)	3,743,599
Coverage (×)	66.7
G + C content (%)	67.3
Completeness (%)	100.0
Contamination (%)	0.48
CDS	3,447
rRNA gene	6
tRNA gene	56

## Data Availability

The raw reads are available in DDBJ Sequence Read Archive under the accession number DRR719414. The complete genome sequence of YSD1 was deposited in DDBJ under the accession number AP043666.
